# Efficacy of traditional Chinese medicine *Cordyceps sinensis* as an adjunctive treatment in patients with renal dysfunction: a systematic-review and meta-analysis

**DOI:** 10.3389/fmed.2024.1477569

**Published:** 2025-01-07

**Authors:** Fenfang Wu, Chunhua Xu, Xinlei Si, Fei He, Kang Xu, Yu Zhang, Shan Lin

**Affiliations:** ^1^Department of Central Laboratory, Shenzhen Hospital, Beijing University of Chinese Medicine, Shenzhen, Guangdong, China; ^2^Department of Nephrology, Longgang Central Hospital of Shenzhen, Shenzhen, Guangdong, China; ^3^School of Life Sciences, Bengbu Medical University, Bengbu, Anhui, China

**Keywords:** *Cordyceps sinensis*, acute kidney injury, inflammation, clinical efficacy, adjunctive treatment

## Abstract

**Objective:**

The effectiveness of using *Cordyceps sinensis* as an adjuvant therapy for patients with renal dysfunction (RD), especially acute kidney injury (AKI), is still a topic of debate. In response to the current conflicting data, the present meta-analysis was conducted to assess the clinical effectiveness of *C. sinensis* in the treatment of RD and to provide evidence for clinical practice.

**Methods:**

Several databases, including PubMed, EMBASE, Cochrane Library, China National Knowledge Infrastructure (CNKI) and Wanfang, were systematically searched updated to March 25, 2024. We used the combined ratio (OR) and diagnostic ratio (DOR) to assess the therapeutic effect of *C. sinensis*. In addition, risk of bias was assessed by Cochrane Risk of Bias Assessment Tool.

**Results:**

The present meta-analysis ultimately incorporated 15 studies comprising a total of 1,310 patients with RD. We pooled estimated the sensitivity, specificity as well as DOR from patient-based analyses with 0.89 (95% confidence interval [CI]: 0.84–0.93), 0.69 (95% CI: 0.59–0.77) and 18.0 (95% CI: 8.0–39.0), respectively. Moreover, we calculated the combined positive likelihood ratio (PLR) as well as negative likelihood ratio (NLR) to be 2.8 (95% CI: 2.1–3.9) and 0.16 (95% CI: 0.10–0.27), respectively. Additionally, area under the curve (AUC) of the summary receiver operating characteristic (SROC) was calculated as 0.88 (95% CI: 0.85–0.90) reflecting prognostic accuracy. Subsequently, subgroup analysis indicated that the clinical efficacy of *C. sinensis* in northern Chinese patients with RD was superior to that of southern. On the other hand, *C. sinensis* significantly reduced patients’ blood creatinine levels, shortened the oliguria period, and increased urine osmolality, indicating it can improve the function of glomeruli and renal tubules.

**Conclusion:**

Our results indicate that *C. sinensis* can be considered a dependable clinical treatment for individuals with RD. It may improve the function of glomeruli and tubules, promote the recovery of tubular function, and thus enhance the clinical therapeutic effects.

**Systematic review registration:**

www.crd.york.ac.uk/PROSPERO/#recordDetails, identifier CRD42024559042.

## Introduction

Renal dysfunction (RD) is a disease with a wide range of duration and progression of kidney deterioration, generally including acute kidney injury (AKI), acute kidney disease (AKD), and chronic kidney disease (CKD). AKI is a multifaceted clinical condition marked by a rapid decline in renal function, and is identified by disruptions in mitochondrial function and the injury of renal tubular cells driven by oxidative stress (1, 2). AKI has high morbidity and mortality rates, and resulted in an enormous economic burden worldwide, seriously affecting the lives and health of patients (3). It is estimated that AKI affects more than 13 million patients worldwide each year and can lead to more than 1.7 million deaths, with common causes such as nephrotoxic damage, renal ureteral obstruction and ischaemia/reperfusion (IR) (4). AKI is defined by a sudden decline in kidney function. If the injury is small and the kidney undergoes adaptive repair, renal function can be fully restored (5). Nevertheless, severe renal damage frequently results in inadequate repair processes, causing disruptions in microcirculation, chronic infiltration of inflammatory cells, loss of renal units, and the development of renal fibrosis. Consequently, individuals with AKI frequently transition to end-stage renal disease (ESKD) and CKD (6). Therefore, it is of great practical significance to explore the potential therapeutic drugs and means to prevent and control the occurrence and progression of RD.

Although there have been many studies on the pathogenesis and diagnosis of AKI in recent years, there have been no major breakthroughs in the prevention and treatment of AKI (7). Currently, treatment of AKI includes correction of reversible causes, maintenance of fluid and electrolyte balance, and renal replacement therapy if necessary (8). Unfortunately, there is no evidence-based medical evidence to show that a particular modern drug is definitively effective against AKI (9). Recently, research on the treatment and prevention of AKI in traditional Chinese medicine (TCM) has made several promising progress (10). Some single-flavored TCMs and their extracts can be used in animal experiments or clinical studies of AKI, and *Cordyceps sinensis* is one of the earlier TCMs used in clinical and animal experiments of AKI (11, 12).

*C. sinensis* (also known as *Ophiocordyceps sinensis*) is a valuable TCM that has been used for centuries as a tonic herb (13). In recent years, *C. sinensis* has been widely used for a variety of diseases, including lung, liver, kidney, cardiovascular system and many others (14). Prior experimental research has demonstrated that *C. sinensis* offers protection against RD in animal models induced by factors such as drug-induced acute tubular toxicity and renal ischemia/reperfusion injury. It has been found to safeguard renal tubules and support their repair, leading to enhanced renal function (15). *C. sinensis* not only has better therapeutic effects in treating non-small cell lung cancer (NSCLC) (16), but it can also significantly improve depressive-like symptoms through immune regulation (17). It is worth noting that *C. sinensis* has significant effects in treating kidney diseases, including diabetic kidney disease (DKD) and AKI (18). The Bailing Capsule, as a major *C. sinensis* preparation, its pharmacological mechanism in treating CKD may be related to immune response and inflammatory response (14), but there are also studies that find The Bailing Capsule can achieve the same effect of treating CKD by regulating the PPARα pathway (19). Previously, several experimental studies have shown that *C. sinensis* has a protective effect on animal models of AKI due to a variety of causes, such as drug-induced acute tubular toxicity, renal ischemia-reperfusion injury, etc., which protects the renal tubules and promotes renal tubular repair and improves renal function (20, 21). While some clinical studies have shown the effectiveness of *C. sinensis* preparations in treating AKI, the majority of these studies are single-center clinical trials with small sample sizes (11). To date, the efficacy and safety of *C. sinensis* preparations for the treatment of RD have not been systematically evaluated. Hence, it is crucial and pressing to conduct a comprehensive analysis of its efficacy and safety.

In this study, we aim to provide credible evidence for the therapeutic efficacy of *C. sinensis* preparations applied to RD through a comprehensive and systematic evaluation of the existing literature, and to investigate whether *C. sinensis* could improve the renal function of patients with RD, promote the recovery of renal tubular function, and thus improve the clinical outcome. Importantly, the conduct of this study will also provide a reference for further research on the action of TCM against RD.

## Methods

### Sources and searches of data

PubMed, EMBASE, Cochrane Library, CNKI and Wanfang, were systematically searched from creation to March 25, 2024. The following search terms were used: (“kidney injury” [Mesh], or “acute kidney injury” [Mesh], or “acute renal failure” [Mesh], or “acute tubular necrosis” [Mesh], or “acute renal injury” [Mesh] or “AKI” [Mesh]), and (“*Cordyceps sinensis*” [Mesh], OR “*Ophiocordyceps sinensis*” [Mesh], OR “*Cordyceps*” [Mesh], OR “*dongchongxiacao*” [Mesh], OR “*dong chong xia cao*” [Mesh], OR “*bailing*” [Mesh], OR “*jinshuibao*” [Mesh] OR “*zhiling*” [Mesh]). This study includes an abstract with a complete results section. A manual check of the bibliography of the retrieved articles was carried out to find additional references. This meta-analysis was conducted in accordance with published PRISMA statements (Preferred Reporting Items for Systematic Reviews and Meta-Analyses) (22, 23). This meta-analysis has been already submitted to PROSPERO with ID 559042.

### Selection of study

We review all citations in order. The full text of relevant articles was searched by title or abstract, and independently reviewed by two researchers (Shan Lin and Chunhua Xu) to identify eligible studies. Differences regarding qualifications were resolved through discussion with the arbitrator (Fengfang Wu). Articles are considered for inclusion that clearly meets the following inclusion criteria. Randomized controlled studies (RCTs) or semi-RCTs evaluating the efficacy and side effects of *C. sinensis* preparations applied to the treatment of RD were included, whether or not blinded, in what language, published or not. Patients with RD who met the diagnostic criteria were included: SCr increased ≥0.3 mg/dL (≥26.5 μmol/L) within 48 h, or SCr increased to 1.5 times the baseline level within 7 d, or urine output was <0.5 mg/kg⋅h for 6 h. Patient inclusion was independent of age, race, gender, and regardless of other comorbidities.

Studies that met the following exclusion criteria were excluded. (1) No randomized controlled trial was conducted; (2) only animal or *in vitro* studies; (3) duplicate published literature; (4) trials not designed with a control group; (5) general reviews or expert reviews; (6) non-clinical trial studies; (7) inaccessibility of full text; and (8) missing records of experimental data.

### Data extraction

Data for each experiment were systematically collected by Shan Lin and Wu Fengfang, respectively. All differences or disagreements between reviewers are evaluated and discussed by the third party reviewer (Xinlei Si) until reached a consensus. The default data of each study included a requirement to recalculate and record the following variables including first author, region or country, year of publication, study design, median age (years), sample size (males), duration of observation, time of inclusion, sensitivity, specificity, area under the curve (AUC; 95% confidence interval [CI]), and reported results from included studies such as C-reactive protein (CRP), serum creatinine (SCr), blood urea nitrogen (BUN) and creatinine clearance rate (Ccr).

### Assessment of quality

In the present study, we used the Cochrane bias risk assessment tool to assess article quality by two independent reviewers (Shan Lin and Chunhua Xu) (24). The tool mainly includes six domains: random sequence generation, allocation concealment, outcome reporting options, blinding method, incomplete outcome data as well as other bias resources. Subsequently, all the six domains were assessed for “applicability issues” as well as “risk of bias,” and each item was judged to be “yes,” “no” or “unclear.”

### Statistic analysis

In the present study, Stata 13.0 (StataCorp, College Station, TX, USA) software was used to statistically analyze the true negative (TN), true positive (TP), false positive (FP) and false negative (FN) rates of each study. The sensitivity, specificity, positive likelihood ratio (PLR) and negative likelihood ratio (NLR) of diagnostic singularity ratio (DOR) were evaluated. A *P*-value of less than 0.05 for the Q statistic and an *I*^2^ value greater than 50% for the *I*^2^ statistic are considered statistically significant heterogeneity (25). A random effects model was applied when heterogeneity was high (*I*^2^ > 50%) (26). Subsequently, the Hardy-Weinberg balance (HWE) of each included study was statistically significant with a *P*-value less than 0.05 by Pearson’s χ^2^ test in the control group (27).

Additionally, in order to evaluate the clinical efficacy of *C. sinensis* in the treatment of patients with RD, we evaluated the AUC as a summary indicator, and plotted the aggregate receiver characteristic curve (SROC) and the aggregate sensitivity and specificity forest plots (28). Subgroup analyses were then also performed by geography or age. Finally, Begg’s and Egger’s tests were used to detect the possible publication bias, and it was considered statistically significant when the *P*-value was less than 0.05 (29).

## Results

### Search for literature

**FIGURE 1:**
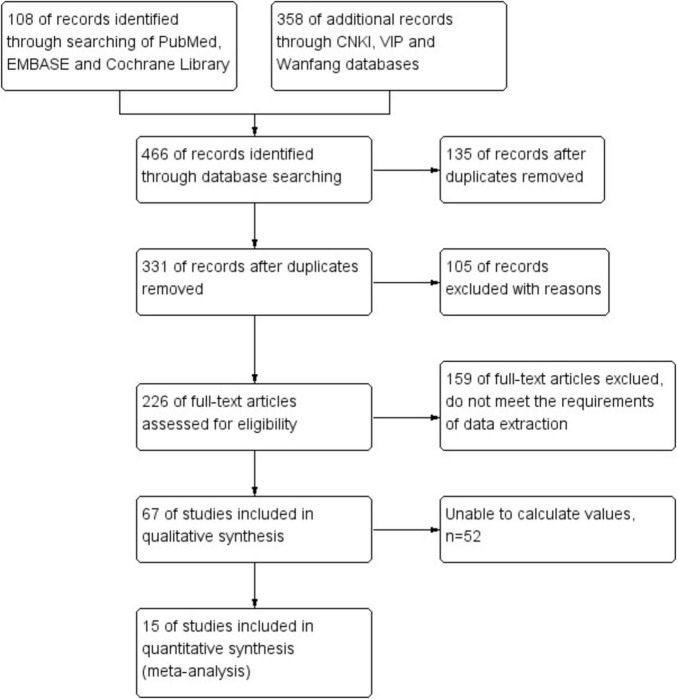
The process of selecting studies for inclusion in this meta-analysis.

Initially, 466 potentially relevant papers were searched in the electronic database, but after screening, 135 papers were systematically eliminated. From the titles and abstracts, 105 studies were clearly not relevant and were ultimately excluded. Subsequently, 159 of full-text articles were certainly excluded because they did not meet the requirements of data extraction. After reviewing the remaining 67 papers, 52 were certainly rejected. In the end, 15 papers were certainly selected. Based on 15 literatures (including 1,310 cases), the efficacy of *C. sinensis* in the clinical treatment of RD patients was systematically evaluated by Meta method. Figure 1 details our step-by-step screening process for included trials.

### The quality and characteristics of the included studies

As shown in Table 1, we systematically extracted basic data to assess the quality of the included trials. Of the 15 trials, six were conducted in North China (30–35) and the remaining 9 were in South China, including 4 in Southeast China (36–39), 3 in South Central China (40–42), and 2 in Southwest China (43, 44). All included studies were from single-center clinical trials between 2004 and 2022. A total of 1,310 patients with RD were enrolled in 15 observational studies, including 255 in North China, 522 in Southeast China, 317 in Central and South China, and 170 in Southwest China. The observation period of the included studies ranged from 4 weeks to 6 months (as shown in Table 1). All studies evaluated the renal function levels of RD patients. Among them, CRP levels were observed in 6 studies and SCr levels were observed in 14 studies, as well as BUN levels were evaluated in 11 studies and Ccr levels were observed in 9 studies, which were shown in Table 2. Additionally, the sensitivity and specificity of the included tests were calculated or given, ranging from 57.10 to 96.08% and from 30.00 to 86.88%, respectively.

**TABLE 1:** Key features of the selected studies.

References	Sample size (male)	Geographic location	Median age	Duration of observation	Enrollment period	Research design
Chai (30)	50 (30)	North China	38 ± 14.00	4–6 months	2005–2008	RCT
Chen (40)	122 (62)	South China	7 ± 1.30	2 weeks	2014.06–2016.01	RCT
Chen (41)	117 (58)	South China	51.4 ± 2.63	2 months	2016.06–2018.04	RCT
He (43)	92 (50)	South China	56.21 ± 9.67	3 months	2018.06–2021.02	RCT
Jin (31)	113 (58)	North China	58.07 ± 10.01	2 weeks	2018.01–2019.07	RCT
Liu (44)	78 (43)	South China	39.25 ± 8.55	1 month	2013.10–2016.10	RCT
Liu (32)	92 (51)	North China	70.55 ± 3.86	8 weeks	2020.06–2021.11	RCT
Lu (33)	102 (59)	South China	63.87 ± 4.80	2 months	2020.01–2021.04	RCT
Quan (42)	72 (39)	South China	NR	4 months	2000–2003	RCT
Tang (34)	106 (56)	South China	46.70 ± 6.43	16 weeks	2019.01–2020.12	RCT
Wu (36)	82 (65)	South China	64.70 ± 5.12	2 weeks	2012.01–2014.01	RCT
Xu (37)	82 (52)	South China	41.8	2 months	2001.04–2003.03	RCT
Xu (35)	52 (25)	South China	7.60 ± 1.58	4 weeks	2016.04–2017.06	RCT
Yang (38)	52 (28)	South China	35.20 ± 8.05	4 weeks	2004.06–2010.06	RCT
Yu (39)	98 (51)	South China	NR	3 months	2011.01–2012.06	RCT

NR, no result.

**TABLE 2:** The reported outcomes of included studies.

References	CRP	SCr	BUN	Ccr	Sensitivity (%)	Specificity (%)	Number of patients			
							**TP**	**FP**	**FN**	**TN**
Chai (30)	NR	√	√	√	66.67	35.00	20	13	10	7
Chen (40)	NR	√	√	NR	91.80	86.88	56	8	5	53
Chen (41)	√	√	√	√	94.82	83.05	55	10	3	49
He (43)	√	NR	NR	NR	89.13	69.57	41	14	5	32
Jin (31)	√	√	NR	√	83.61	67.31	51	17	10	35
Liu (44)	NR	√	NR	√	92.31	76.92	36	9	3	30
Liu (32)	NR	√	√	√	95.65	80.43	44	9	2	37
Lu (33)	√	√	√	√	96.08	84.31	49	8	2	43
Quan (42)	NR	√	√	NR	84.21	47.06	32	18	6	16
Tang (34)	√	√	√	NR	92.45	81.13	49	10	4	43
Wu (36)	NR	√	√	NR	95.12	53.66	39	19	2	22
Xu (37)	NR	√	√	NR	57.10	30.00	24	28	18	12
Xu (35)	NR	√	√	√	80.80	69.20	21	8	5	18
Yang (38)	NR	√	NR	NR	92.30	65.40	24	9	2	17
Yu (39)	√	√	√	√	89.80	69.39	44	15	5	34

NR, no result; CRP, C-reactive protein; SCr, serum creatinine; BUN, blood urea nitrogen; Ccr, creatinine clearance rate.

### Methodological quality and publication bias

**FIGURE 2:**
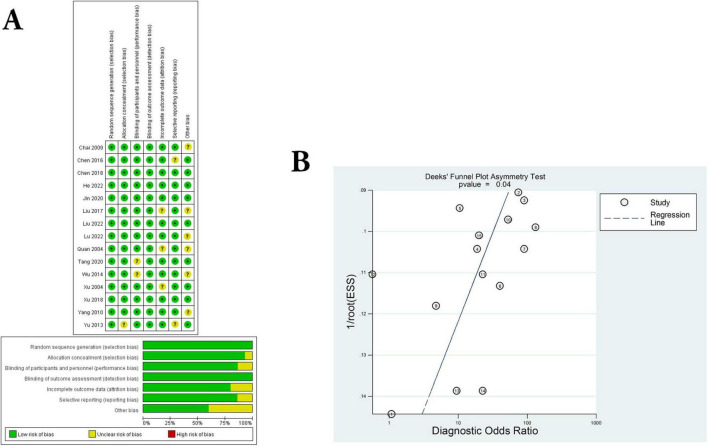
Assessment of literature quality and adjunctive therapeutic effect of *C. sinensis*. **(A)** Quality assessment of included eligible studies by the Cochrane Risk of Bias Assessment Tool. **(B)** Forest plot of Deeks funnel plot of the efficacy of *C. sinensis* as an adjunctive treatment in patients with RD.

In the present study, all trials have detailed inclusion criteria as well as exclusion criteria for patients. Subsequently, the quality of each included study was assessed using the Cochrane risk of Bias tool (all studies had a Cochrane score of 10 or above). In addition, we averaged the overall quality of the included studies. The results of the Cochrane assessment can be clearly seen in Figure 2A, and the source of “uncertain risk” maybe mainly due to low-quality small samples, and poor quality of experimental method design.

Next, the funnel plot shown in Figure 2B evaluates publication bias, showing no significant threshold effects or significant asymmetries. In other words, there was no significant publication bias in the present meta-analysis. Consequently, the results of the present study do not appear to change significantly due to which have not yet been certainly published.

### Assessment of the clinical effectiveness of *C. sienesis*

**FIGURE 3:**
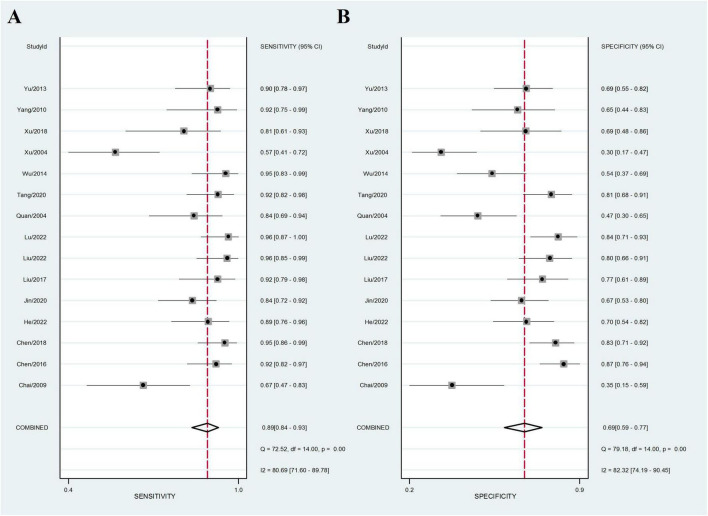
Forest plot of the efficacy of *C. sinensis* as an adjunctive treatment in patients with RD. **(A)** The sensitivity of the efficacy of *C. sinensis* as an adjunctive treatment in patients with RD. **(B)** The specificity of the efficacy of *C. sinensis* as an adjunctive treatment in patients with RD.

**FIGURE 4:**
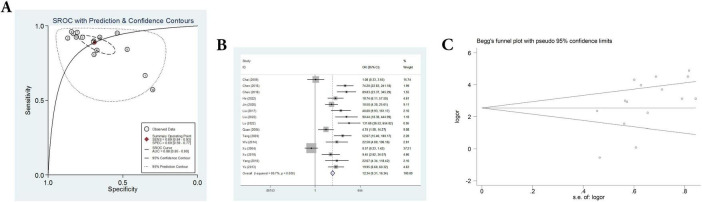
A comprehensive assessment of the efficacy and reliability of *C. sinensis* as an adjunctive treatment in patients with RD. **(A)** SROC with prediction and confidence contours. **(B)** Forest plot of the efficacy of *C. sinensis* as an adjunctive treatment in patients with RD. **(C)** The Begg’s funnel plot for testing publication bias of the efficacy of *C. sinensis* as an adjunctive treatment in patients with RD.

Fifteen sets of data were extracted from 15 qualified literatures which were shown in Table 2, certainly including sensitivity, specificity, AUC, 95% CI, as well as TP, FN, FP, TN, etc. Fifteen studies evaluated the clinical efficacy of *C. sinensis* as a potential clinical treatment for RD patients, with a total of 1,310 RD patients. The aggregated data from the above trials were summarized in Table 2. In the evaluation of the clinical efficacy of *C. sinensis*, its pooled sensitivity was calculated as 0.89 (95% CI: 0.84–0.93) (Figure 3A), the specificity was calculated as 0.69 (95%CI 0.99–0.77) (Figure 3B), the positive likelihood ratio (PLR) was calculated as 2.8 (95%CI 2.1–3.9), as well as the negative likelihood ratio (NLR) was calculated as 0.16 (95%CI, 0.10–0.27). Furthermore, it had a certain diagnostic probability ratio (DOR) of 18 (95% CI 8–39) according to the random effects model. Additionally, the AUC for the predictive SROC accuracy was calculated to be 0.88 (95% CI 0.85–0.90; Figure 4A). Figure 4B summarizes the clinical efficacy of *C. sinensis* in the treatment of RD patients. It is therefore not difficult to draw the following conclusions from the results that *C. sinensis* has a significant clinical effect on RD, and *C. sinensis* is a reliable clinical treatment for RD patients.

### Subgroup analysis for geographic positions and outcomes

To further understand the differences in the clinical efficacy of *C. sinensis* for patients with RD in different geographic positions and with different outcomes, a systematic subgroup analysis was carried out whose results were shown in Table 3. The clinical efficacy of *C. sinensis* in patients with RD showed significant differences according to the comparison of DOR and AUC. Subsequently, in the subgroup analysis of geographic positions, the DOR as well as AUC of clinical efficacy of *C. sinensis* in north China were significantly higher than those in south China (DOR, 20; AUC, 0.88 vs. DOR, 16; AUC, 0.87), indicating that the clinical efficacy of *C. sinensis* in northern Chinese patients with RD was superior to that of southern Chinese patients with RD, and may have better performance in recurrence and poor prognosis. The reason may be related to the geographical environment and dietary habits of the northern population. In the subgroup analysis of outcomes, the clinical efficacy of *C. sinensis* for RD patients with outcome of Ccr was significantly better than that for RD patients with outcome of BUN (DOR, 24; AUC, 0.89 vs. DOR, 17; AUC, 0.88) and Scr (DOR, 24; AUC, 0.89 vs. DOR, 17; AUC, 0.88), whereas significantly worse than compared with that for RD patients with outcome of CRP (DOR, 24; AUC, 0.89 vs. DOR, 35; AUC, 0.91).

**TABLE 3:** Subgroup analysis of geographic position, sample size, median age and outcomes.

Studies	Number		Sensitivity	Specificity	PLR	NLR	DOR	AUC
Geographic positions	6	North China	0.89 (0.79–0.94)	0.72 (0.58–0.83)	3.2 (1.9–5.3)	0.16 (0.07–0.35)	20 (6–72)	0.88 (0.85–0.91)
	9	South China	0.89 (0.82–0.94)	0.67 (0.54–0.77)	2.7 (1.8–4.0)	0.16 (0.08–0.32)	16 (6–46)	0.87 (0.84–0.90)
Sample size	5	>100	0.87 (0.79–0.92)	0.61 (0.49–0.71)	2.2 (1.6–3.1)	0.22 (0.11–0.41)	10 (4–27)	0.81 (0.78–0.85)
	10	<100	0.92 (0.87–0.95)	0.81 (0.74–0.87)	4.9 (3.4–7.0)	0.10 (0.05–0.17)	51 (22–117)	0.93 (0.91–0.95)
Median age	6	>50	0.93 (0.87–0.96)	0.74 (0.65–0.82)	3.6 (2.5–5.1)	0.10 (0.06–0.18)	36 (15–86)	0.93 (0.90–0.95)
	7	<50	0.85 (0.73–0.92)	0.66 (0.49–0.80)	2.5 (1.4–4.5)	0.23 (0.10–0.52)	11 (3–42)	0.84 (0.81–0.87)
Outcomes	6	CRP	0.91 (0.63–0.79)	0.76 (0.69–0.82)	3.9 (2.9–5.2)	0.11 (0.07–0.19)	35 (16–73)	0.91 (0.88–0.93)
	14	SCr	0.89 (0.83–0.93)	0.69 (0.58–0.77)	2.8 (2.0–4.0)	0.16 (0.09–0.28)	17 (7–41)	0.88 (0.85–0.90)
	11	BUN	0.89 (0.82–0.93)	0.68 (0.55–0.79)	2.8 (1.8–4.3)	0.16 (0.08–0.32)	17 (6–50)	0.88 (0.84–0.90)
	8	Ccr	0.90 (0.82–0.94)	0.73 (0.63–0.81)	3.3 (2.2–4.9)	0.14 (0.07–0.27)	24 (9–65)	0.89 (0.86–0.91)

Meanwhile, in the subgroup analysis of sample size, the DOR as well as AUC of clinical efficacy of *C. sinensis* in sample size less than 100 were significantly higher than those in sample size more than 100 (DOR, 51; AUC, 0.93 vs. DOR, 10; AUC, 0.81), indicating that the clinical efficacy of *C. sinensis* in small sample size was superior to that of large sample size. Furthermore, in the subgroup analysis of median age, the clinical efficacy of *C. sinensis* for RD patients with median age more than 50 was significantly better than that for RD patients with median age less than 50 (DOR, 36; AUC, 0.93 vs. DOR, 11; AUC, 0.84).

Interestingly, based on the above subgroup analysis results, *C. sinensis* has excellent clinical efficacy for RD patients in high-latitude areas of China, which might be related to the high RD incidence in this area. At the same time, *C. sinensis* showed the best clinical efficacy in RD patients with CRP results, which may indicate that *C. sinensis* can achieve satisfactory clinical efficacy by improving immune indicators (such as CRP) in RD patients. In addition, *C. sinensis* shows better treatment efficacy in elderly RD patients and groups with small sample sizes.

### Analysis of publication bias and heterogeneity

In addition, heterogeneity analysis and SROC analysis were also performed. No “shoulder-arm” pattern was found in the SROC space (Figure 4A), indicating no threshold effect. Furthermore, Begg’s funnel plot was applied to evaluate the probability of publication bias and the comprehensive results, as shown in Figure 4C, indicate that the probability of publication bias is very low.

Additionally, heterogeneity in the fifteen included studies was assessed by meta-regression. The results showed that the differences of heterogeneity test are statistically significant (*I*^2^ = 86.7%, *P* = 0.000), suggesting that that there is significant heterogeneity between the included 15 studies. Subsequently, we adopted the exclusion method to rank the factors that may cause heterogeneity, and found that when three papers were excluded, the heterogeneity was satisfactory (*I*^2^ = 47.1%, *P* = 0.036), indicating the reason for the heterogeneity is the inclusion of these 3 studies. After systematic analysis, it was believed that the source of heterogeneity may be due to differences in medication and baseline population characteristics.

### Sensitivity and adverse reaction

To investigate the effect of a single dataset on the combined DOR, each study in this meta-analysis was deleted each time. Subsequently, the results of the sensitivity analysis indicated the robustness of the results of the present study (Figure 3A).

In the literature included above, none of the side effects were explicitly mentioned in the treatment of RD with *C. sinensis* preparations, and no studies have reported on the occurrence complications of *C. sinensis* preparations. Moreover, the safety of *C. sinensis* preparations was clinically confirmed to be high with few side effects.

## Discussion

Acute kidney injury is an important risk factor for the development and progression of RD (45). AKI has become a global public health issue, with approximately 1.7 million deaths per year worldwide due to AKI and its complications. Even mild AKI may have adverse consequences and may progress to renal fibrosis, which is the end result of all end-stage renal disease (46). Therefore, exploring the potential mechanism of RD, especially AKI, and searching for new therapeutic agents are of great practical significance in preventing and controlling the occurrence and progression of AKI.

Previously, RD was mostly treated with western medicines (47). Western symptomatic treatment can temporarily relieve the symptoms, correct the water electrolytes, and maintain the balance of acidity in the body, but it cannot completely stop the progression of renal failure (48). At present, the prevention and treatment of RD with TCM has become a research hotspot, and the effect of combining TCM with western medicine is better (49). With the characteristics of multi-targets, multi-pathways, low toxicity and low price, TCM has certain advantages in the prevention and treatment of RD (10). It has been shown that “*Dahuanggancao*” decoction can significantly reduce blood creatinine and urea nitrogen levels and improve renal pathological changes in AKI mice, thus slowing down the progression of AKI (50). In another study, *Panax ginseng* saponins were shown to be able to reduce blood creatinine, blood urea nitrogen as well as cystatin C in AKI mice while also improving renal histopathology and tubular cell apoptosis, and reducing tubular injury and mitochondrial dysfunction in AKI (51). Chinese medicine *C. sinensis* preparation, whose main ingredient is the mycelium of artificially fermented *C. sinensis*, and whose main active ingredients include cordyceps polysaccharides, cordyceps acid, and alkaloids, which can effectively enhance the immune system, and play the functions of hypoglycemia, cardiovascular protection, anti-lipid peroxide, as well as anti-inflammation, and protection of liver and kidney functions, etc (52). Recently, studies have been conducted on *C. sinensis* originating from locations outside of China, analyzing the nucleic acids and polysaccharides and other bioactive components of *C. sinensis* collected from Bhutan, and it was found that it is a reasonable substitute for natural *C. sinensis* and can help improve its performance in the fields of health and pharmaceutical food (53). Generally, *C. sinensis* preparations can exert their pharmacological effects through immune regulation, and some studies have found that *C. sinensis* can alleviate liver inflammation and fibrosis induced by CCl4 by promoting the activation of liver NK cells, showing significant anti-liver fibrosis effects (54). There have also been reports of *C. sinensis* having a protective effect on radiation-induced immune suppression, which may be related to its anti-apoptosis action and regulation of adaptive immunity (55). Therefore, the therapeutic effects of *C. sinensis* preparations in treating kidney diseases such as DKD and AKI through immune regulation are also worth further investigation.

The results of this study showed that after the treatment of RD by *C. sinensis* preparation, the Scr and BUN levels of the patients were lower than those of the control group, indicating that *C. sinensis* preparation can delay the development of renal failure and improve the immune function. Modern pharmacological studies have shown that cordyceps polysaccharides, cordycepin and other active substances contained in *C. sinensis* are effective immunomodulatory preparations with a wide range of immunopharmacological effects, which can improve the immune function of the body, which may be one of the main reasons for the efficacy of *C. sinensis* preparations (56). It was shown that *C. sinensis* preparations can promote fatty acid oxidation and inhibit fat synthesis by regulating the PPARα pathway, thus reducing the accumulation of triglycerides in rat kidney and repairing the damage of renal function (19). Other studies have also found that *C. sinensis* preparations can prevent RD by inhibiting perforin expression in NK cells, as well as can mitigate renal injury by reducing the expression level of pro-inflammatory factors through the NF-κB pathway (12). In addition, the combination of *C. sinensis* preparation and western medicine can complement each other, take into account the specimen, and improve the kidney function through different mechanisms of action, enhance immunity, and delay the progression of the disease (20). In this study, it was found that in the process of RD treatment using *C. sinensis* preparations, while improving renal function, it also reduces the level of inflammation and improves the microinflammatory state. Meanwhile, the immunological indexes of the patients in the observation group after the use of the drug were significantly lower than those of the control group, which fully demonstrated that the *C. sinensis* preparations have a positive significance in promoting the restoration of renal function, delaying the progression of nephropathy, and improving the microinflammatory state.

In recent years, the application of *C. sinensis* preparations in the field of nephropathy has received increasing attention from researchers and clinicians (57). This study is the first to systematically evaluate the adjuvant therapeutic effect of *C. sinensis* preparations in patients with acute kidney injury. There are also some limitations in this study. First, *C. sinensis* preparations are mostly used in mainland China, and sample sizes for clinical studies and methodological limitations in study design are more common. Second, the inclusion of fewer studies and small sample sizes implies a higher risk of random error. Third, for the reasons of funnel plot is not completely symmetric, it is believed that the causes may be publication bias, and poor methodological quality of smaller studies. Finally, all of the included studies had short follow-up periods, and it is not clear whether the short-term favorable effects can be maintained and leads to favorable outcomes in the long term. Therefore, future clinical studies with longer follow-up periods are needed to determine long-term clinical effects, as well as larger sample sizes and multicenter randomized clinical studies are also needed to provide strong evidence.

## Conclusion

*Cordyceps sinensis* preparations have significant clinical efficacy in RD, and can be reliable clinical therapeutic agents for patients with RD. Especially, *C. sinensis* preparations have shown excellent efficacy in RD when used as adjunctive therapies in combination with western drugs. Nevertheless, well-designed and larger studies are needed to be further and fully clarified the clinical efficacy of *C. sinensis* in adjunctive treatment of patients with RD.

## Data Availability

The original contributions presented in this study are included in this article/Supplementary material, further inquiries can be directed to the corresponding author.
